# Cosmetically Applicable Soluble Agonists for Toll-like Receptor 2 Produced by Fermentation of Asparagus Extract Supplemented with Skimmed Milk Using *Lactobacillus delbrueckii* subsp. *lactis* TL24 Consist of Molecules Larger than 100 kDa and Can Be Stabilized by Lyophilization with Dextrin

**DOI:** 10.3390/molecules29194557

**Published:** 2024-09-25

**Authors:** Yasuhiko Komatsu, Kanako Matsunaga

**Affiliations:** Development Research Department, Snowden Co., Ltd., 3-7-16 Iwamoto-cho, Chiyoda-ku, Tokyo 101-0032, Japan; k_matsunaga@snowden.co.jp

**Keywords:** lactic acid bacterium, *Lactobacillus delbrueckii* subsp. *lactis*, asparagus, Toll-like receptor 2, keratinocytes, tight junction, dextrin

## Abstract

Cosmetically applicable soluble agonists for Toll-like receptor 2 (TLR2), which can strengthen skin barrier function, were produced by fermentation of asparagus (*Asparagus officinalis* L.) extract supplemented with skimmed milk using *Lactobacillus delbrueckii* subsp. *lactis* TL24. Their molecular size was estimated to be >100 kDa. Their TLR2-stimulating activity was stable over 1 year at 4 °C, but it decreased by more than 95% within 10 and 4 months at 25 °C and 40 °C, respectively. The possibility of stabilization of TLR2-stimulating activity by powdering was tested, and we found that lyophilization with 10% or a higher amount of dextrin could stabilize the activity even at 40 °C. The powdered fermented product dose-dependently stimulated TLR2. It augmented the formation of tight junctions in normal human keratinocytes, as detected by fluorescence staining of occludin and ZO-1, whereas their protein and gene expression levels did not increase, suggesting that a change in subcellular localization of these proteins without significant changes in their amounts might be responsible. The powder nature has some benefits over the aqueous, besides stability, e.g., it can be dissolved just before application, allowing fresh material to be used each time, and it may widen a range of cosmetic applications in non-aqueous types of cosmetics.

## 1. Introduction

Maintaining moisture of the skin is an important function of skincare cosmetics, because loss of moisture brings about not only visible and tactile changes, but also alteration in skin’s sensory components, which is the cause of so-called dry skin symptoms, including dryness and various discomforts [1]. Two main types of approach exist to attain this goal: physical and biological. Physical approaches include humectants, emollients, and occlusives [1,2]. Humectants, such as hyaluronic acid and glycerol, are moisturizers that gather water from the atmosphere and/or deeper layers of the skin. Emollients, such as ceramides and dimethicone, are moisturizers that can help soften and smoothen the skin by filling gaps in skin cells. Occlusives, such as petroleum jelly, cover the skin surface to prevent evaporation of moisture. One of the important biological approaches is to tighten cell-to-cell junctions via stimulation of epidermal keratinocytes to prevent evaporation of moisture from the skin and also to prevent harmful effects of adverse environmental conditions. Stimulation of Toll-like receptor 2 (TLR2) of epidermal keratinocytes has been reported to augment tight junctions (TJs) between cells to strengthen the barrier function of a cell sheet [3,4]. Since such a strengthening of TJs is thought to increase the skin barrier function, it might be promising to apply agonists for TLR2 in cosmetics. However, in their studies, as the agonists for TLR2, either peptidoglycan prepared from pathogenic *Staphylococcus aureus* or the synthetic ligand Pam3CSK4 was used. Therefore, no direct application for cosmetics had been expected.

To overcome such a drawback, we produced cosmetically applicable soluble TLR2 agonists by fermentation with *Lactobacillus delbrueckii* subsp. *lactis* TL24 (TL24) of appropriate plant extracts supplemented with skimmed milk [5]. Extracts of plants, such as edible stem of asparagus (*Asparagus officinalis* L.), are good sources to be used for fermentation. TL24 was selected from 184 strains of lactic acid bacteria isolated from various fermented foods worldwide for its ability to produce high amounts of soluble TLR2 agonists, and the combination of asparagus extract, and skimmed milk was found to be more efficient in producing soluble TLR2 agonists than either one alone [5]. A previous study reported that the fermented product of asparagus extract supplemented with skimmed milk by TL24 (LAB-Asp) specifically stimulated TLR2 and strengthened the barrier function of the cell sheet of normal human epidermal keratinocytes [5]. For practical applications of such fermented products in cosmetics, information regarding the molecular nature and stability of the active components may be important. However, such data have not yet been published. In this study, we estimated the molecular sizes of the active components and examined their stability in aqueous solutions. Unfortunately, the TLR2-stimulating activity of the fermented product was not stable after a month even at moderate temperatures, although it withstood heat inactivation at 110 °C for 20 min at the last step of fermentation. Therefore, we attempted to stabilize its activity by lyophilization with water-soluble dextrin; this process was successful. Furthermore, biological activities related to cosmetic applications of the lyophilized fermented product were examined.

In this study, we tried to elucidate the molecular nature of the active components of LAB-Asp to stimulate TLR2, and found that they were larger than 100 kDa, not highly stable in aqueous solution, but could be stabilized by lyophilization with water-soluble dextrin. The lyophilized fermented products with dextrin successfully activated TLR2 after solubilization and augmented TJs between normal human keratinocytes. This tightening of TJs were not associated with the increase in ZO-1 and occludin but changes in their subcellular localization.

## 2. Results and Discussion

### 2.1. Chemical Properties of Asparagus Extract and Its Fermented Product

Table 1 and Table 2 show the chemical properties of asparagus extract and its fermented product. In Table 1, the result of the analysis of free amino acids in asparagus extract, asparagus extract supplemented with skimmed milk (after sterilization), and the fermented product with TL24 (LAB-Asp) are shown. Consistent with previous studies of amino acid contents in asparagus [6,7], the asparagus extract contained large amounts of Asn, Glu, and Gln. Although Gln disappeared after the addition of skimmed milk followed by sterilization and did not reappear after the fermentation, Asn and Glu were maintained at a higher level even after fermentation. Gamma-aminobutyric acid (GABA) has been reported as a characteristic amino acid in asparagus [7,8], and GABA was also found both in asparagus extract and its fermented product in this study. Some amino acids (phosphor-serine, Thr, Gly, alpha-aminobutyric acid, Val, Ile, Phe, and ornithine) appeared by fermentation, and Asp, Ser, Glu, GABA, and Pro increased by fermentation. These results suggest that LAB-Asp contains some amino acids characteristic of asparagus and also contains other amino acids increased and/or appearing due to fermentation with TL24.

Table 2 shows some other chemical properties of asparagus extract and its fermented product (LAB-Asp). Since two characteristic polyphenols in asparagus are rutin and its aglycon, quercetin [9,10], we tried to detect them. In the asparagus extract used in this study, approximately 50 µg/mL rutin was detected. However, we could not detect quercetin. This might be because the hydrophobic nature of quercetin made it difficult to be extracted by hot water in this study. Since rutin disappeared after fermentation, biological activities of this molecule will not be expected in LAB-Asp. By fermentation, pH lowered, lactic acid largely increased, and glucose present in asparagus extract was consumed by fermentation, undetected in LAB-Asp.

Although some low molecular weight compounds in asparagus extract that survive fermentation, or those increased or appearing due to fermentation, might have beneficial effects in the cosmetic application of LAB-Asp, e.g., GABA for inhibition of melanogenesis and induction of type I collagen gene expression [11,12], lactic acid and Orn as natural moisturizing factors [13,14,15],we will focus on the TLR2-stimulating activity of LAB-Asp in the following sections.

### 2.2. Estimation of Molecular Size of the TLR2-Activating Components

**Figure 1:**
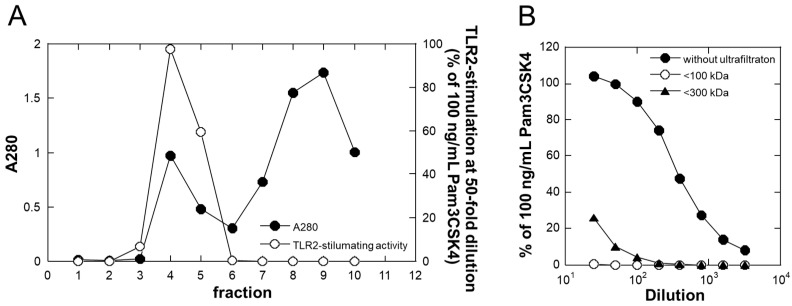
Estimation of molecular size of TLR2 stimulants in LAB-Asp. (**A**) Gel-filtration chromatogram using a PD-10 column. After the column was equilibrated with PBS, 1 mL of LAB-Asp was applied and eluted with PBS to obtain 1 mL fractions. A280 (closed circles) and TLR2-stimulation activity (open circles) were determined and plotted. (**B**) Ultrafiltration analysis of LAB-Asp. TLR2-stimulating activities of LAB-Asp without ultrafiltration (closed circles), passed through molecular weight cutoff of 100 kDa (open circles) and 300 kDa (closed triangles) were plotted. Each result is a representative of at least 3 experiments.

Figure 1A shows a gel-filtration chromatogram of LAB-Asp using a PD-10 column. As shown in this figure, components possessing TLR2-stimulating activity were eluted in the void fractions, indicating that the active components were larger than 5000 Da. Next, the TLR2-stimulating activity of the effluents of ultrafiltration was examined using molecular weight cut-offs of 100 and 300 kDa. As shown in Figure 1B, no activity was detected in the effluent that passed through the 100 kDa membrane, whereas small amounts of activity were detected in that passing through the 300 kDa membrane. This result indicated that the active components consisted of molecules larger than 100 kDa. Since TLR2 is a receptor stimulated by cell wall components of gram-positive bacteria, such as lactic acid bacteria, the active components in LAB-Asp might have been shed from the cell walls of TL24 as soluble large-molecular-weight structures. The high molecular weight nature of the active components in LAB-Asp is thought to be suitable for cosmetics because the risk concerning penetration into deeper skin structures and stimulation of immune cells is low.

### 2.3. Effect of Lytic Enzyme for Lactic Acid Bacteria on the TLR2-Activating Components

**Figure 2:**
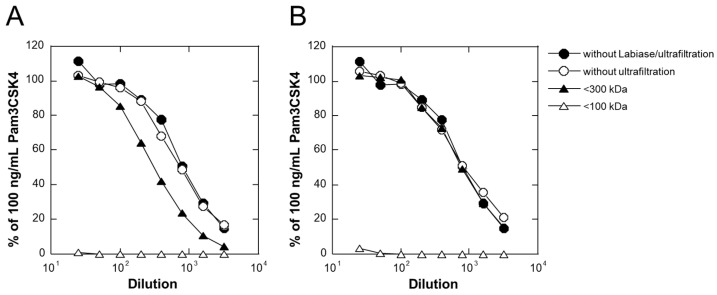
Ultrafiltration analysis of LAB-Asp after the treatment with Labiase at (**A**) 2.5 mg/mL and (**B**) 10 mg/mL at 37 °C for 1 h. TLR2-stimulating activities of LAB-Asp itself without Labiase treatment or ultrafiltration (closed circles). Labiase-treated LAB-Asp without ultrafiltration (open circles), passed through a molecular weight cutoff of 100 kDa (open triangles) and 300 kDa (closed triangles) were plotted. Each result is a representative of at least 3 experiments with similar results.

Since the TLR2-activating components in LAB-Asp might be thought to be derived from the cell wall of TL24, the effect of Labiase, a lytic enzyme preparation for lactic acid bacteria isolated from *Streptomyces fulvissimus* TU-6, (Ozeki Corporation, Hyogo, Japan) [16] on TLR2-stimulating activity of LAB-Asp was examined. As shown in Figure 2, TLR2-stimulating activity between 100 kD and 300 kD increased with the treatment of LAB-Asp with Labiase in a dose-dependent manner (compare closed triangles in Figure 1B and Figure 2A,B), suggesting that a part of the active components was susceptible to Labiase and their molecular size was reduced to some extent (<300 kDa), but maintained at >100 kDa. However, since the overall activity was maintained even after the treatment with Labiase, the TLR2-stimulating activity itself might be thought to survive against the cell wall lysing activity of Labiase, which consists of β-N-acetyl-D-glucosaminidase, muramidase, and endopeptidase activities [16]. The elucidation of the detailed molecular structure of the active components in LAB-Asp, consisting of a mixture of molecules of large size (>100 kDa), will be an important but difficult task for the future.

### 2.4. Stability of TLR2-Activating Activity and Its Stabilization by Lyophilization with Dextrin

**Figure 3:**
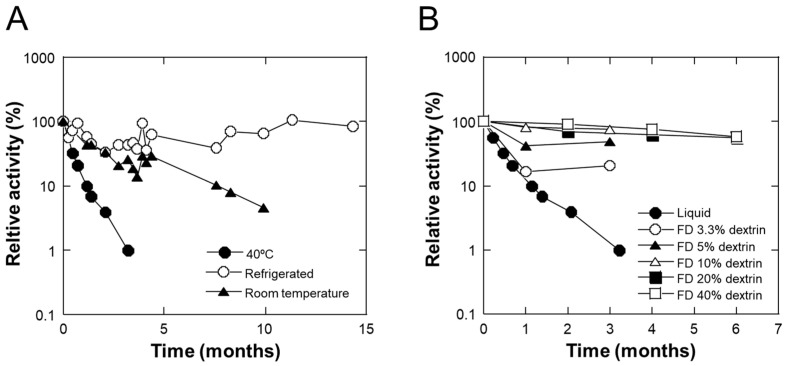
Stability of TLR2-stimulating activity of LAB-Asp and its freeze-dried powder prepared with dextrin. (**A**) Time-dependent change in TLR2-stimulating activity of LAB-Asp at 40 °C (closed circles), under refrigerated conditions (approximately 4 °C; open circles), and at room temperature (approximately 25 °C; closed triangles), indicating that the stability was lower at the higher temperature. (**B**) Time-dependent change in TLR2-stimulating activity at 40 °C of freeze-dried powders prepared using various amounts of dextrin (open circles, closed triangles, open triangles, closed squares, and open squares for 3.3, 5, 10, 20, and 40% of dextrin, respectively). For comparison, the activity of the liquid sample used in (**A**) is also plotted in the same figure.

To use these materials in cosmetics, their stability in aqueous solutions is important. Because sterilization at a high temperature (110 °C) was applied at the final step of fermentation, it was assumed that there would be no stability issues. However, as shown in Figure 3A, the TLR2-stimulating activity of LAB-Asp decreased at 25 °C (a decrease of 95.5% over 9.9 months) and at 40 °C (a decrease of 99.0% over 3.2 months), although it remained stable for more than 1 year at 4 °C. This may reflect the nature of the high molecular weights of the active components, as shown in Figure 1. This suggests that it is difficult to use LAB-Asp in aqueous cosmetics per se because of shelf-life problems.

Some cosmetic ingredients, such as vitamin C, are unstable in aqueous environments. One of the appropriate ways to use components that are unstable in aqueous solutions in cosmetics might be to dissolve the dried powder just before application. In fact, stabilization by microencapsulation with excipients/wall materials by spray- or freeze-drying of unstable components, such as vitamin C and phenolic compounds, has been widely applied [17,18,19,20], and such types of powered cosmetics are available in the market. To examine the possible use of LAB-Asp in a powdered form in cosmetics, we lyophilized LAB-Asp using excipients. The easily soluble excipient, dextrin NSD300, was tested at various concentrations, and it was found that lyophilization of LAB-Asp with 10% or higher amounts of NSD300 could effectively stabilize the TLR2-stimulating activity even at 40 °C (Figure 3B). By keeping a sufficiently safe margin, the lyophilized powder with 40% NSD300 (LAB-Asp-FD) was chosen for further experiments. As the process used to prepare powdered LAB-Asp retained its biological activities in our study, it might be possible to use other technologies, including spray drying, which can be addressed in the future.

### 2.5. Activation of HEK Blue hTLR2 and THP-1 Cells via TLR2 by LAB-Asp-FD

**Figure 4:**
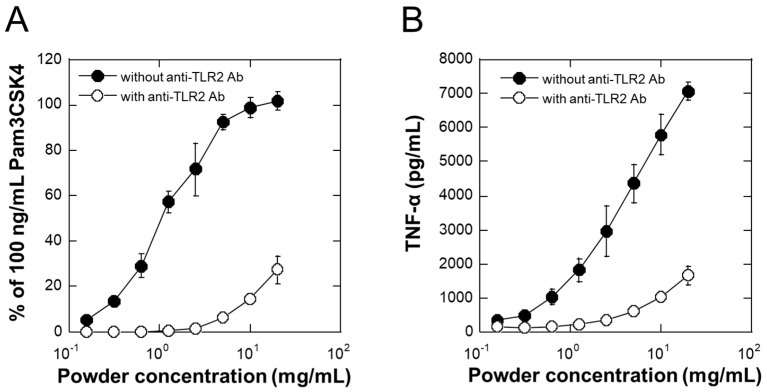
Biological activities of freeze-dried powder of the fermented product prepared using 40% dextrin (LAB-Asp-FD). (**A**) TLR2-stimutating activity was determined using HEK Blue hTLR2 reporter cells after incubation for 24 h at various concentration of LAB-Asp-FD with (open circles) or without (closed circles) 1 µg/mL of anti-hTLR2 antibody. (**B**) Concentrations of TNF-α in culture supernatants of THP-1 cells after incubation for 24 h at various concentration of LAB-Asp-FD with (open circles) or without (closed circles) 1 µg/mL of anti hTLR2 antibody. Each point represents mean ± S.D. of 3 wells.

Because it is important to elicit biological activities even after lyophilization, we examine these points in the following two sections. Figure 4A shows dose-dependent activation of TLR2 in HEK Blue hTLR2 cells by LAB-Asp-FD, indicating that concentrations of 10 mg/mL or higher are sufficient to fully activate TLR2 in this cell system. Figure 4B shows dose-dependent induction of the production of TNF-α in differentiated THP-1 cells, suggesting that it can activate macrophages. Since both the activations were largely inhibited in the presence of anti-hTLR2 antibody (Figure 4A,B), these effects were thought to be mediated through the activation of TLR2.

Although unnecessary activation of macrophages might promote undesirable inflammatory effects, the large size of active components in LAB-Asp-FD will limit such adverse effects if preventing application on inflammatory sites.

### 2.6. Augmentation via TLR2 of TJs in Keratinocytes by LAB-Asp-FD

**Figure 5:**
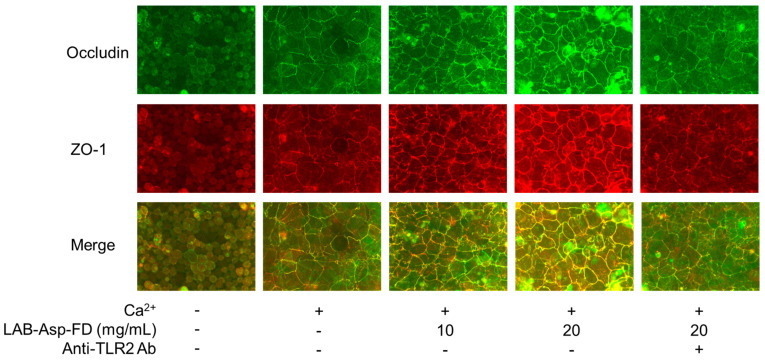
Effect of LAB-Asp-FD on tight junctions (TJs) in normal human epidermal keratinocytes. Normal human keratinocytes were treated with 1.8 mL CaCl_2_ for 3 days in the presence or absence of LAB-Asp-FD (10 or 20 mg/mL), and then the TJ proteins (occludin and ZO-1) were stained with specific antibodies conjugated with fluorescent dyes (Alexa Fluor™ 488 and 555, respectively), and immunofluorescence images were captured. To evaluate the extent of TLR2-signaling, 1 µg/mL of anti-hTLR2 antibody was added to 20 mg/mL LAB-Asp-FD. The images for occludin (green), ZO-1 (red), and their merged examples are shown, indicating dose-dependent augmentation of TJs by LAB-Asp-FD and their inhibition in the presence of anti-hTLR2 antibody. “+” and “-” denote “presence” and “absence”, respectively. This result is a representative of at least 3 experiments.

The most important biological effect of LAB-Asp-FD for application in cosmetics may be the augmentation of TJs between keratinocytes. To examine whether LAB-Asp-FD can augment TJs between keratinocytes, normal human epidermal keratinocyte cells were treated with LAB-Asp-FD for 3 days, and then TJ proteins (occludin and ZO-1) were stained with specific antibodies conjugated with fluorescent dyes, and immunofluorescence images were obtained. As shown in Figure 5, addition of CaCl_2_ induced the formation of TJ structures reflecting induction of differentiation by Ca^2+^ ions, and coexistence with LAB-Asp-FD augmented the TJ structure in a dose-dependent manner. The effect of 20 mg/mL LAB-Asp-FD in augmenting TJs was inhibited by 1 µg/mL anti-hTLR2 antibody. These results suggest that LAB-Asp-FD could augment TJs in keratinocytes even after lyophilization with dextrin and that signaling via TLR2 plays an important role in this augmentation.

### 2.7. Quantitative Reverse-Transcription Polymerase Chain Reaction and Western Blotting Analyses of TJ Proteins in Normal Human Epidermal Keratinocytes

To determine whether the augmentation of TJs by LAB-Asp-FD was accompanied by an increase in the amount of TJ proteins, gene expression and protein levels were examined by quantitative reverse-transcription polymerase chain reaction (RT–qPCR) and western blotting (WB), respectively.

**Figure 6:**
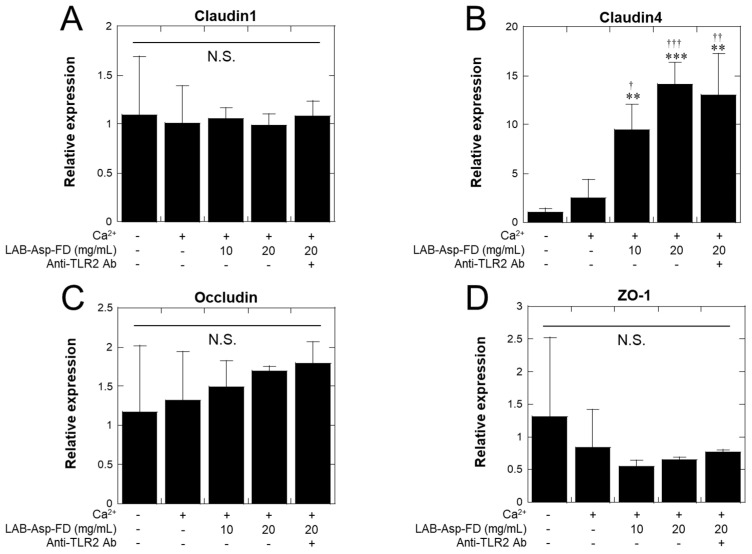
Effects of LAB-Asp-FD on the expression of genes encoding tight junction proteins. Confluent normal human epidermal keratinocytes were treated with 1.8 mL CaCl_2_ for 24 h in the presence or absence of LAB-Asp-FD (10 or 20 mg/mL). To evaluate the extent of TLR2-signaling, 1 µg/mL of anti-hTLR2 antibody was added to 20 mg/mL LAB-Asp-FD. After total RNA was extracted, reverse-transcription and quantitative PCR were performed. The extents of gene expression of (**A**) claudin1, (**B**) claudin 4, (**C**) occludin, and (**D**) ZO-1 compared to those in the absence of Ca^2+^, LAB-Asp-FD, and anti-TLR2 antibody were calculated using the 2^−ΔΔCt^ method with β-actin as an internal standard. Each bar represents mean with SD of 3 culture wells. “+” and “-” denote “presence” and “absence”, respectively. ** *p* < 0.01 and *** *p* < 0.001 vs. “no Ca^2+^, no LAB-Asp-FD, and no anti-TLR2 Ab”; ^†^ *p* < 0.05, ^††^ *p* < 0.01, and ^†††^ *p* < 0.001 vs. “1.8 mM Ca^2+^, no LAB-Asp-FD, no anti-TLR2 Ab”. N.S. denotes no significant differences between groups.

**Figure 7:**
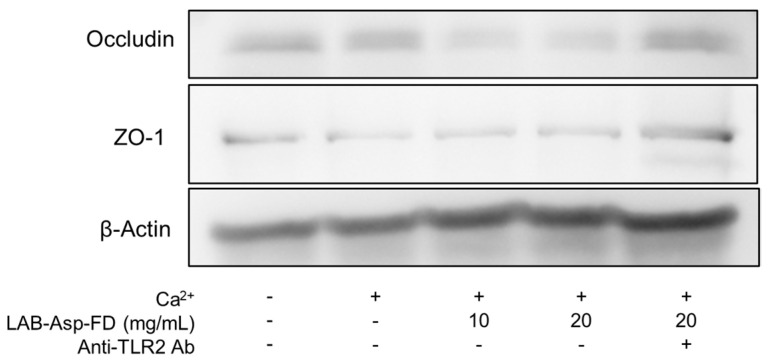
Western blot analyses of occludin and ZO-1 proteins. Confluent normal human epidermal keratinocytes were treated with 1.8 mL CaCl_2_ for 72 h in the presence or absence of freeze-dried powder of the fermented product prepared with 40% dextrin (LAB-Asp-FD) (10 or 20 mg/mL). To evaluate the extent of TLR2-signaling, 1 µg/mL of anti-hTLR2 antibody was added to 20 mg/mL LAB-Asp-FD. After 5 µg/lane of total proteins were separated using 10% SDS-PAGE and transferred to PVDF membrane, the amount of each protein was detected using corresponding specific mouse monoclonal antibodies followed by HRP-labeled secondary antibody. β-actin was used as a standard. “+” and “-” denote “presence” and “absence”, respectively. This result is a representative of at least 3 experiments.

In the RT–qPCR analysis, as shown in Figure 6, no significant changes were observed in the claudin-1 (Figure 6A), occludin (Figure 6C), or ZO-1 (Figure 6D) genes, whereas gene expression of the claudin-4 gene was significantly increased by treatment with LAB-Asp-FD compared to treatment both with and without Ca^2+^ controls (Figure 6B). However, no inhibition of the increased expression of the claudin-4 gene by anti-hTLR2 antibody was observed. Possible implications of the up-regulation of claudin-4 gene will be discussed in 2.8. As depicted in Figure 7, WB analysis showed no clear increase in the amounts of occludin and ZO-1 proteins because of the addition of Ca^2+^ and also of LAB-Asp-FD, and no decrease in their amounts was observed upon the addition of anti-hTLR2 antibody.

### 2.8. Possible Mechanisms Underlying the Augmentation of TJs by LAB-Asp-FD

As shown in Figure 5, the TJs between keratinocytes detected by immunostaining of occludin and ZO-1 proteins were augmented by the addition of LAB-Asp-FD. Since the effect of LAB-Asp-FD was inhibited by the presence of anti-hTLR2 antibody, it is reasonable to assume that the stimulation of TLR2 by LAB-Asp-FD is responsible for this augmentation. However, no increase in the expression of genes encoding occludin, ZO-1, or claudin-1 was observed following LAB-Asp-FD treatment (Figure 6). As shown in Figure 7, the levels of occludin and ZO-1 proteins did not increase after treatment with LAB-Asp-FD. Therefore, it can be assumed that the observed augmentation of TJs occurred without an increase in the levels of these proteins. In other words, the augmentation of TJs was mainly due to changes in subcellular localization of these proteins.

Although the mechanisms underlying the effects of LAB-Asp-FD on TJs remain to be elucidated, some mechanisms may be speculated. One possible mechanism is the activation of atypical protein kinase C (aPKC). Yuki et al. [3] and Helfrich et al. [21] reported the role of aPKCs activated by calcium ions and/or bacterial peptidoglycans in strengthening the TJs and transepithelial electrical resistance (TER) in keratinocytes without increasing the amount of TJ proteins. However, in their studies, although the inhibition of aPKCζ/ι by specific inhibitors brought about a reduction of TER, no apparent disintegration of TJ proteins from the TJ structure was observed. Another possibility might be the activation of RhoA GTPase via activation of TLR2, since the activation of RhoA by integrin-linked kinases has been reported to be indispensable for keratinocyte differentiation and epidermal barrier function, including the assembly of TJ proteins [22], and a pathway that activates RhoA by stimulating TLR2 is reported [23]. The other possibility might be related to claudin-4. Kubo et al. reported that, in human keratinocytes, stimulation of toll-like receptor 3 (TLR3), a pattern recognition receptor other than TLR2, suppressed the expression of ΔNp63, a p53 family transcription factor, which in turn brought about enhanced expression of claudin-4 [24]. They also reported that stimulation of TLR2 by insoluble zymosan slightly enhanced the expression of claudin-4 without apparent suppression of the expression of ΔNp63 [24]. Since Aono and Hirai reported that phosphorylation of claudin-4 by aPKC is required for tight junction formation in a human keratinocyte cell line [25], it is possible that the elevated expression of claudin-4 by LAB-Asp-FD via TLR2 increased the amount of phosphorylated claudin-4 without activating aPKC itself, which in turn tightened the TJs.

Although some speculations like the above are possible, lack of detailed data related to the intracellular signaling from TLR2 stimulation to the augmentation of TJs is a limitation of this study. Further studies are necessary to elucidate the underlying mechanisms.

### 2.9. Risks and Benefits of the Application of LAB-Asp-FD in Cosmetics

Stimulation of TLR2 has a potential risk of activating immune cells, including macrophages, to promote inflammation. However, the large molecular nature (>100 kDa) of the active components in LAB-Asp-FD is thought to limit such a risk to some extent, since most immune cells rarely exist in the epidermis of healthy skin [26]. In fact, the safety of LAB-Asp-FD for cosmetic application was guaranteed by a primary skin irritation test (alternative method; conducted at Kirei Testing Labo, Tokyo, Japan), eye irritation test (alternative method; conducted at RiSaRaOPT Corporation, Osaka, Japan), 24 h occlusive patch test (conducted at Kirei Testing Labo), and Repeated Insult Patch Test (RIPT; conducted at Eurofins CRL Cosmetics Inc., Chicago, IL, USA) at 25 mg/mL. Therefore, application of LAB-Asp-FD at 25 mg/mL or lower concentration can be thought to be safe. Of course, as with many other cosmetic products, it is important to prevent application to already inflamed sites.

In this study, we conducted lyophilization of LAB-Asp with water-soluble dextrin to stabilize its TLR2-stimulating activity. However, the powder nature of LAB-Asp-FD has some benefits over aqueous LAB-Asp, besides stability. One benefit of LAB-Asp-FD is that it can be dissolved just before application, allowing fresh material to be used each time. Other benefits may include a wider range of cosmetic applications. That is, while application of aqueous LAB-Asp in non-aqueous type of cosmetics (e.g., loose powder, lip gloss, lip stick, lip balm, creamy foundation, eye shadow, etc.) is limited, LAB-Asp-FD might be possible to be used in such applications.

## 3. Materials and Methods

### 3.1. Lactic Acid Bacterium

The lactic acid bacterium TL24 was kindly provided by Dr. Taku Miyamoto, Professor Emeritus at the Okayama University, Okayama, Japan. TL24 was cultivated in De Man, Rogosa, and Sharpe medium at 37 °C to obtain a seed culture that was used for the fermentation of asparagus extract supplemented with skimmed milk.

### 3.2. Mammalian Cells

As normal human epidermal keratinocytes, we used NHEK(NB) cells (Kurabo, Osaka, Japan). These cells were cultivated in DermaLife K keratinocyte medium (Lifeline Cell Technology, Frederick, MD, USA) at 37 °C under 5% CO_2_ atmosphere in a humidified chamber. All the experiments were conducted using cells passaged four or fewer times.

As TLR2 reporter cells, we used HEK Blue hTLR2 cells (InvivoGen, Toulouse, France) according to the manufacturer’s instruction. HEK Blue hTLR2 cells were cultivated in Dulbocco’s modified Eagle’s medium supplemented with selective antibiotics (HEK Blue selection, InvivoGen), penicillin, streptomycin, normocin, and 10% fetal bovine serum, as described in a previous study [5], at 37 °C under 5% CO_2_ atmosphere in a humidified chamber.

As a human monocyte-like cell line, we used THP-1 cells, which were purchased from JCRB Cell Bank (National Institutes of Biomedical Innovation, Health and Nutrition, Osaka, Japan). THP-1 cells were cultivated in RPMI 1640 medium supplemented with penicillin, streptomycin, and 10% fetal bovine serum at 37 °C under 5% CO_2_ atmosphere in a humidified chamber.

### 3.3. Production of Fermented Product Having TLR2-Stimulating Activity and Its Lyophilization

Lactic acid bacteria-fermented asparagus extract (LAB-Asp), which has TLR2-stimulating activity, was produced as described previously, with minor modifications [5]. In brief, one part of asparagus (*Asparagus officinalis* L.) edible stem was mixed with two parts of purified water, and the extraction was conducted at 95 °C for 1 h. The asparagus extract produced was mixed with 3% skimmed milk (Takanashi Milk Products, Kanagawa, Japan), heat sterilized (110 °C, 20 min), 2% seed culture of TL24 was added, and fermented at 37 °C for 24 h under gentle stirring. After sterilization at 110 °C for 20 min, LAB-Asp was obtained by filtration.

The lyophilized LAB-Asp powder was produced as follows: LAB-Asp was mixed with various amounts of water-soluble dextrin (NSD300; San-ei Scrochemical, Aichi, Japan) and lyophilized under vacuum with a freeze-drier TF5-80TNNN (Takara Seisaku-sho, Tokyo, Japan). LAB-Asp containing water-soluble dextrin was frozen at −50 °C and then dried under vacuum (10 Pa or lower) at 20 °C for over 12 h. The dried cake thus obtained was milled and sieved to yield the lyophilized powder of LAB-Asp.

### 3.4. Chemical Analyses of Asparagus Extract and Its Fermented Product

Analysis of free amino acid concentration was performed with an automated amino acid analyzer JLC-500/v2 (JEOL Ltd., Tokyo, Japan). Concentrations of rutin and quercetin were determined by using an ultra-performance liquid chromatography (UPLC) system (ACQUITY UPLC H-Class, Waters, Milford, MA, USA) equipped with a reverse-phase column (ACQUITY UPLC HSS T3 Column, 100 Å, 1.8 µm, 2.1 mm × 100 mm). The elution condition is as follows: buffer A, 0.1% formic acid; buffer B, acetonitrile containing 0.1% formic acid; flow rate, 0.2 mL/min; A:B = 85:15 (0 to 4.5 min), 85:15 to 0:100 (4.5 to 11 min); 0:100 (11 to 14 min); 0:100 to 85:15 (11 to 14 min); 85:15 (14 to 17 min); detection at 350 nm. Samples were extracted with Sep-Pak C18 Plus Short Cartridge (Waters), eluted with methanol, and injected into the column. The amounts of rutin and quercetin were determined by comparing the peak areas of samples with standards: rutin (Nacalai tesque, Kyoto, Japan), quercetin (Fujifilm Wako, Osaka, Japan). L- and D-lactic acids were quantified by using a lactic acid assay kit (Megazyme, Bray, Ireland). Glucose concentration was determined with a glucose sensor (Care Fast R; NIPRO, Osaka, Japan).

### 3.5. Treatment of LAB-Asp with Labiase

As the enzyme that can lyse the cell wall of lactic acid bacteria, Labiase (Ozeki Corporation, Hyogo, Japan) was used [16]. LAB-Asp was mixed with various amount of Labiase and incubated at 37 °C for 1 h, and then incubated at 80 °C for 1 h to inactivate the enzyme activity.

### 3.6. TLR2-Stimulating Activity

TLR2-stimulating activity was determined using HEK Blue hTLR2 reporter cells according to a previously described method [5]. As a positive control, 100 ng/mL of Pam3CSK4 (InvivoGen) was used. To examine the role of TLR2 in stimulatory activity, anti-human TLR2 (hTLR2) antibody (Clone 383936; R&D Systems, Minneapolis, MN, USA) was used to block the signaling via TLR2. The TLR2-stimulating activities of the samples were expressed as relative values (%) of absorbance at 655 nm (A655), reflecting reporter activity of the sample compared to that of the positive control placed in the same culture plate.

When analyzing the stability of the TLR2-stimulating activities of the samples, the amount of each sample necessary to attain 50% activity of the positive control was compared to the value at time = 0. In the first step, fold-dilution of aqueous samples and concentration of the powder samples required to attain 50% activity of the positive control were determined. Since the necessary amounts of aqueous and powdered samples to attain 50% activity of the positive control are proportional to “fold-dilution” and “inverse of concentration”, respectively, the stabilities of samples were evaluated by comparing these values at each time point to that at time = 0 (considered as100%).

### 3.7. TNF-α Production from THP-1 Cells

THP-1 cells were seeded in each well of a 96-well culture plate in 100 µL (2 × 10^5^ cells/mL) and cultivated for 2 days. Addition of 200 nM phorbol 12-myristate 13-acetate (PMA; Fujifilm Wako) induced THP-1 cells to develop into macrophages. After cultivation for 2 days, the medium was replaced with fresh medium without PMA. After another cultivation period of 3 days, the test samples were applied. To examine the role of TLR2, an anti-hTLR2 antibody (R&D Systems) was used to block the signaling via TLR2. Next, after another day of cultivation, the plate was transferred to −80 °C to stop the reaction and maintained at this temperature until the assay was performed.

Concentrations of TNF-α in culture supernatants were determined using the Amplified Luminescence Proximity Homogeneous assay (Alpha) technology [27].TNF-α (Human) HP Immunoassay kit (PerkinElmer, Shelton, CT, USA) employing AlphaLISA beads was used according to the manufacturer’s instructions. In brief, standard solutions of TNF-α and samples were mixed with a mixture of anti-TNF-α antibody-labeled acceptor beads and biotinylated anti-TNF-α antibody, incubated for 1 h under shaking. Next, streptavidin-coated donor beads were added, and the plate was incubated for 30 min under shaking. Signals were detected using the NIVO microplate reader (PerkinElmer), and TNF-α concentrations in the samples were calculated using a standard curve.

### 3.8. Gel-Filtration and Ultrafiltration

A PD-10 desalting column (Cytiva, Tokyo, Japan) was used to roughly estimate whether the active components were larger than 5000 Da. After the column was equilibrated with phosphate-buffered saline (PBS), 1 mL of the fermented product was applied, and 1 mL fractions were eluted with PBS. Absorbance at 280 nm (without dilution) and TLR2-stimulating activity (after 50-fold dilution) of each fraction were determined.

Ultrafiltration by centrifugation was performed using Amicon Ultra (molecular weight cut-off = 100 kDa; Merck Millipore, Burlington, MA, USA) and Nanosep Omega (molecular weight cut-off = 300 kDa; Pall, Port Washington, NY, USA), and TLR2-activating activities of the effluents were determined.

### 3.9. Immunofluorescence Staining of TJ Proteins

NHEK(NB) cells (1.2 × 10^5^ cells/mL) were seeded in 1 mL of medium in each well of a 24-well cell culture plate and cultivated for 4 days. The medium was replaced with fresh medium containing the test samples as follows. To induce differentiation of NHEK(NB) cells to form a permeability barrier [28,29], 1.8 mM CaCl_2_ was added. In the presence of CaCl_2_, 10 or 20 mg/mL LAB-Asp-FD was added to examine the effects of the powdered fermented products. To assess whether the effects of the powdered fermented products were produced via TLR2, 1 µg/mL anti-hTLR2 antibody (R&D Systems) was added to 20 mg/mL LAB-Asp-FD.

After cultivation for 3 days, the cell sheet was washed thrice with 1 mL PBS, fixed with cold ethanol (1 mL) for 30 min at 4 °C for 30 min, washed thrice with 1 mL PBS, and permeabilized with 1 mL PBS containing 0.1% Triton X-100 for 10 min at 25 °C. The cell sheet was then washed thrice with 1 mL TBST (150 mM NaCl, 100 mM Tris, and 0.1% Tween 20, pH8) and blocked with 3% bovine serum albumin (BSA) and 3% polyvinylpyrrolidone (PVP) K-30 in TBST at 25 °C for 1 h. After washing the cells thrice with 1 mL TBST, 1 mL of solution containing anti-occludin and anti-ZO-1 antibodies conjugated with Alexa Fluor™ 488 and 555, respectively, (Thermo Fisher Scientific, Waltham, MA, USA) in TBST containing 0.1% bovine serum albumin were added and incubated at 4 °C overnight. After extensive washing five times with TBST under shaking, green (occludin) and red (ZO-1) fluorescence images were captured using a digital fluorescence micrometer BZ-810 (Keyence, Osaka, Japan) equipped with ×20 objective lens.

### 3.10. Quantitative Reverse-Transcription Polymerase Chain Reaction (RT–qPCR)

NHEK(NB) cells were seeded and treated with the test samples, similar to that for TJ analysis, and cultivated for 1 day. After washing thrice with 1 mL PBS, total RNA was extracted using the RNeasy Plus Micro Kit (Qiagen, Hilden, Germany) according to the manufacturer’s instructions. The extracted total RNA was subjected to Reverse-Transcription (RT) using PrimeScript RT Master Mix (Takara Bio, Shiga, Japan), and the resulting DNA was used as a template for quantitative real-time polymerase chain reaction (qPCR) with TB Green Premix Ex Taq II (Tli RNaseH Plus) (Takara Bio) using the Thermal Cycler Dice Real-time System II (Takara Bio) by employing a 2 step method (40 cycles of 95 °C for 5 sec and 57 °C for 30 sec). The primers used to perform the RT–qPCR are listed below: 5′-TTGACTCCTTGCTGAATCTGAG-3′ and 5′-TTCTGCACCTCATCGTCTTC-3′ for claudin-1; 5′-CTCTGCGAACGTTAAGTCCG-3′ and 5′-AATGTTGCTGCCGATGAAGG-3′ for claudin-4; 5′-GCAAAGTGAATGACAAGC GG-3′ and 5′-GACCTTCCTGCTCTTCCCTT-3′ for occludin; 5′-CCAGCATCATCAACCTCTGC-3′ and 5′-CATGCGACGACAATGATGGT-3′ for ZO-1; 5′-CTGTGGCATCCACGAAACTA-3′ and 5′-AGCTCAGGCAGGAAAGACAC-3′ for β-actin. Relative expression of each gene relative to the sample with no additive to the medium was evaluated by the 2^−ΔΔCT^ method using β-actin as an internal standard [30].

### 3.11. Western Blotting (WB)

NHEK(NB) cells were seeded and treated with test samples similar to that for TJ analysis and cultured for 4 days. After washing thrice with 1 mL PBS, the cell sheet was lysed with 4% sodium dodecyl sulfate (SDS) in 125 mM Tris-HCl, pH6.8, and viscosity of the lysate was lowered by passing it through a syringe needle. Five micrograms of proteins thus obtained were subjected to 10% SDS-polyacrylamide gel electrophoresis and transferred to a polyvinylidene fluoride membrane. After blocking the membrane with 3% BSA and 3% PVP K-30 in TBST for 1 h, occulin, ZO-1, and β-actin were detected using 2, 2, and 0.5 µg/mL of mouse monoclonal antibodies: 33-1500 (Thermo Fisher Scientific), 33-9100 (Thermo Fisher Scientific), and sc-47778 (Santa Cruz Biotechnology, Dallas, TX, USA), respectively. The antibodies were dissolved in TBST containing 0.1% BSA (TBST-BSA) and incubated for 30 min under shaking. After washing 5 times with TBST for 3 min, the membrane was incubated with the secondary antibody, HRP-conjugated anti-mouse IgG (Cytiva) diluted 1/2500 with TBST-BSA, for 30 min under shaking. After washed 5 times with TBST for 3 min, protein bands were detected by chemiluminescence method using the Amersham ECL Prime Western Blotting Detection kit (Cytiva) with LuminoGraph I (ATTO, Tokyo, Japan).

### 3.12. Statistics

Statistical analysis between test groups was performed using Tukey’s honestly significant difference method, and differences were considered statistically significant if the probability (P) was less than 0.05.

## 4. Conclusions

This study was conducted to elucidate the molecular nature of the active components of cosmetically applicable agonists for TLR2 produced by fermentation of asparagus extract supplemented with skimmed milk using a lactic acid bacterium TL24 (LAB-Asp). Molecular size of active components of LAB-Asp were estimated to be larger than 100 kDa, which is an advantageous property to be applied in cosmetics that do not stimulate inflammation. Since the molecular size of the active components decreased with the treatment with Labiase, a lytic enzyme preparation for lactic acid bacteria, the active components were thought to be derived from the cell wall structure of TL24 and shed as large soluble molecules. Although its TLR2-stimulating activity was stable over 1 year at 4 °C, it decreased significantly both at 25 °C and at 40 °C, suggesting that its application as a solution in cosmetics is limited. However, stabilization of the TLR2-stimulating activity of LAB-Asp was possible by lyophilization with 10% or a higher amount of water-soluble dextrin to be stable for 6 months even at 40 °C. The powdered fermented product (LAB-Asp-FD) produced using lyophilization with dextrin dose-dependently stimulated TLR2-reporter cells, in addition to the production of TNF-α in THP-1 cells. It augmented the formation of tight junctions in normal human epidermal keratinocytes, as detected by fluorescence staining of occludin and ZO-1, whereas the protein and gene expression levels did not increase, suggesting that the change in the subcellular localization of these proteins without large changes in their amounts might be responsible. The powder nature of LAB-Asp-FD has some benefits over aqueous LAB-Asp beside stability. One benefit of LAB-Asp-FD is that it can be dissolved just before application, allowing fresh material to be used each time. Other benefits may include a wider range of cosmetic applications in non-aqueous types of cosmetics.

## Figures and Tables

**Table 1:** Free amino acid analyses of asparagus extract and its fermented product.

Amino acid ^(1)^	Free Amino Acid Concentration (nmol/mL) ^(2)^			Fold-Increase by Fermentation ^(3)^
	Asparagus Extract	Asparagus Extract Supplemented with Skimmed Milk (After Sterilization)	After Fermentation and Sterilization (LAB-Asp)	
P-Ser	N.D.	N.D.	51.2	+
Asp	181	131	342	2.61
Thr	131	N.D.	130	+
Ser	510	268	446	1.66
Asn	3611	3155	2490	0.789
Glu	796	687	1139	1.66
Gln	1080	N.D.	N.D.	−
Gly	194	N.D.	211	+
Ala	807	671	93.6	0.139
a-ABA	N.D.	N.D.	2.2	+
Val	83.4	N.D.	158	+
Ile	N.D.	N.D.	113	+
Leu	57.9	N.D.	190	+
Phe	N.D.	N.D.	9.72	+
GABA	581	286	366	1.28
Orn	N.D.	N.D.	43.7	+
Lys	61.0	N.D.	N.D.	−
Pro	481	299	560	1.87

^(1)^ Amino acids are described in three letter code. P-Ser, a-ABA, GABA, and Orn denote phospho-serine, alpha-aminobutyric acid, gamma-aminobutyric acid, and ornithine, respectively. ^(2)^ N.D. denotes “not detected”. ^(3)^ Ratio of the concentration after fermentation to that before fermentation. “−”, and “+” denote “N.D. both before and after fermentation” and “appeared by fermentation”, respectively.

**Table 2:** Some chemical properties of asparagus extract and its fermented product.

Items	Asparagus Extract	After Fermentation and Sterilization (LAB-Asp)
Polyphenol		
Rutin (µg/mL)	49.5 ± 21.0 (n = 4)	N.D. (n = 1)
Quercetin (µg/mL)	N.D. (n = 1)	N.D. (n = 1)
pH	5.85 ± 0.17 (n = 7)	3.49 ± 0.05 (n = 12)
Lactic acid		
L-lactic acid (mg/mL)	0.0800 ± 0.0052 (n = 3)	0.150 ± 0.088 (n = 7)
D-lactic acid (mg/mL)	0.0367 ± 0.0174 (n = 3)	7.94 ± 0.82 (n = 7)
Glucose (mg/dL)	320 ± 61 (n = 7)	N.D. (n = 12)

Means ± standard deviations are depicted, where numbers of independent samples assayed are described in parentheses. N.D. denotes “not detected”.

## Data Availability

All data are included in the paper.
